# Prognostic Factors Affecting the Outcome of Surgical Root Canal Treatment—A Retrospective Cone-Beam Computed Tomography Cohort Study

**DOI:** 10.3390/jcm13061692

**Published:** 2024-03-15

**Authors:** Salma AlKhuwaitir, Shanon Patel, Abdulaziz Bakhsh, John Spencer Rhodes, Luis Miguel Ferrández, Francesco Mannocci

**Affiliations:** 1 Department of Endodontics, King Saud Medical City Dental Hospital, Riyadh 12746 361, Saudi Arabia; s.alkhuwaitir@ksmc.med.sa; 2 Department of Endodontics, Centre of Oral Clinical & Translational Sciences, Faculty of Dentistry, Oral & Craniofacial Sciences, King’s College London, London WC2R 2LS, UK; shanonpatel@gmail.com; 3 Department of Restorative Dentistry, Division of Endodontics, Faculty of Dentistry, Umm Al-Qura University, Makkah 24382, Saudi Arabia; aabakhsh@uqu.edu.sa; 4 The Endodontic Practice, 15 Penn Hill Avenue, Poole BH14 9LU, UK; john@rootcanals.co.uk (J.S.R.); luis@rootcanals.co.uk (L.M.F.)

**Keywords:** endodontics, apicoectomy, apical periodontitis, cone-beam computed tomography, treatment outcomes, mineral trioxide aggregate, Biodentine, prognosis

## Abstract

**Aim:** To assess the association between demographic and clinical variables and the outcome of root-end surgery using digital periapical radiographs (PA) and cone-beam computed tomography (CBCT). **Methodology:** One hundred and fifty teeth that received endodontic microsurgery were clinically and radiographically examined (PA and CBCT scans) after 1 and 2 years. Two calibrated endodontists evaluated the radiographic healing based on a six-point outcome classification. The outcome was classified using both strict (healed) and loose (healing) criteria. The success rates were calculated, and several outcome prognostic factors were assessed. **Results:** One hundred and fifty teeth were assessed with both radiographic systems. When “loose” success criteria were applied using PA, 90% (n = 135) of teeth were assessed as having had a favourable outcome (90%; 95% CI: 85.2–94.8%), whereas 90.7% (n = 136) of teeth showed a successful outcome when assessed with CBCT (90.7%; 95% CI: 86.0–95.3%). When “strict” success criteria were applied, there was a statistically significant difference (*p* = 0.018) between the success rates of mineral trioxide aggregate (MTA) (63.7%) and Biodentine (95.5%). **Conclusions:** Within the limitations of this study, endodontic microsurgery showed a high success rate. Among all the demographic and clinical variables assessed, in the multiregression analysis, only the use of Biodentine was associated with a higher proportion of “complete” healings compared to MTA or Intermediate Restorative Material (IRM) when assessed using CBCT. MTA and Biodentine performed similarly when “incomplete” healings were regarded as successful outcomes.

## 1. Introduction

The aim of endodontic treatment is the prevention and/or elimination of apical periodontitis through chemo-mechanical debridement of the root canal system [1,2,3]. Persistent and/or re-infection may result in the failure of root canal treatment [4].

Surgical endodontic treatment (SET) is usually indicated to manage apical periodontitis (AP) when orthograde (re-)root canal treatment is not viable [5,6].The success rates of contemporary SET, i.e., using magnification, micro-instruments, ultrasonic tips, and contemporary root-end filling materials, have been reported to be over 90% [7].

It is well established that periapical radiographs (PR) have limitations when assessing radiographic signs of AP and, therefore, can overestimate the resolution of AP [8,9,10]. To overcome such limitations, CBCT has been developed and managed to produce three-dimensional images of the tooth and the surrounding structures [11,12]. CBCT’s increased sensitivity may result in the detection of AP, which with PA may appear as complete or partially resolved [13,14]. PA radiographs, however, remain in the daily practice, and are the method of choice for the assessment of the outcome of surgical endodontics due to their availability and low radiation exposure for the patient. To the authors’ knowledge, there is no published CBCT study assessing the outcome of root-end surgery using different retrofilling materials.

The aim of this study was to assess the association between several clinical variables and the outcome of root-end surgery using PA and CBCT; the variables taken into consideration are reported in Table 1.

## 2. Materials and Methods

### 2.1. Ethical Approval

The protocol of the study was approved by the London—Riverside Research Ethics Committee (REC reference: 20/LO/0024). The participants were given detailed verbal and written information regarding the purpose of the study, and written consent was obtained in accordance with the Declaration of Helsinki.

### 2.2. Study Design

This retrospective cohort study included patients who underwent endodontic microsurgery for the treatment of apical periodontitis in the endodontic postgraduate unit at Guy’s Hospital and The Endodontic Practice private clinic located in Poole, England between January 2019 and December 2021. Patients attending the standard 1-year recall radiographic and clinical review following surgical endodontics were included, and consent was obtained to take a CBCT scan of the treated teeth at 1 and 2 years, in addition to the periapical radiographs that are routinely taken.

Clinical and radiographic data for each patient were reviewed, and suitable candidates for the study were chosen with signs of AP that were managed with SET. Exclusion criteria included the following: patients less than 18 years old, pregnant patients, probing depths > 3 mm, perio-endo lesions, and clinical and/or radiographic signs of perforations and/or fractures. The STROBE (Strengthening the Reporting of Observational Studies in Epidemiology) checklist and statement (Supplementary Materials) were followed [15].

### 2.3. Periapical Microsurgery Procedure

All SET procedures were performed at Guy’s and St. Thomas’ Foundation Trust, London, UK, and in The Endodontic Practice private clinic located in Poole, England, by either specialist endodontists or endodontic postgraduate students supervised by specialist endodontists using a standardised surgical protocol.

SET was performed with the aid of a dental operating microscope (3-step entree; Global, St Louis, MO, USA). The patient was asked to rinse with a chlorhexidine mouthwash (Corsodyl, GlaxoSmithKline, Middlesex, UK), and local anaesthetic Lidocaine and/or Articaine was administered. A full-thickness flap papilla preservation flap was reflected, and an osteotomy and root-end resection was performed with a long diamond bur attached to a high-speed surgical handpiece NSK Ti-MAX 450 L (Nakanishi International, Kanuma, Japan). Granulomatous tissue was curetted and biopsied for histopathological analysis. Root-end resection was performed perpendicular to the long axis of the root with a long diamond bur attached to a high-speed surgical handpiece with a 45-degree-angle head and rear air exhaust.

All retrograde preparations were 3 mm deep and prepared using an ultrasonic tip (Acteon, Norwich, UK). The cavity was filled with either mineral trioxide aggregate (ProRoot, MTA Sirona Dentsply, Baillagues, Switzerland), Biodentine (Septodont, Saint-Maur-des-Fossés, France), Intermediate Restorative Material (IRM Dentsply, York, PA, USA) or bioceramic putty (Totalfill BC, FKG, La Chaux-de-Fonds, Switzerland). The flap was repositioned and reapproximated using 5-0 Ethilon sutures (Ethicon, Somerville, NJ, USA). Post-operative instructions were given to the patient, and sutures were removed within one week.

### 2.4. Review Appointments

Patients were recalled at 1- and 2-year review appointments. At each recall visit, an intraoral examination was undertaken, including percussion, palpation, periodontal probing, and mobility.

### 2.5. Radiographic Technique

PR radiographs and CBCT scans were taken pre-operatively and at the one- and two-year follow-up appointments. All radiographs at Guy’s Hospital were taken using a paralleling technique for standardisation with a beam-aiming device X-ray unit (Heliodent, Sirona, Bensheim, Germany) and photostimulable phosphor plates (Digora Optime; Soredex, Tuusula, Finland); the exposure parameters for the periapical radiographs were 66 kV, 7.5 mA and 0.10 s. Small-volume (40 mm^3^) CBCT scans were taken for the area of interest using 3D Accuitomo F170 (J Morita Manufacturing, Kyoto, Japan) with exposure parameters of 90 kV, 5.0 mA, and 17.5 s.

For the patients treated in private practice, a paralleling technique was also adopted when periapical radiographs with a beam-aiming device X-ray unit (Acteon, Mérignac, France) were taken. Intraoral sensors from Planmeca ProScanner (Planmeca OY, Helsinki, Finland) were used with exposure parameters of 70 kV, 0.61, and 0.32 s. Small-volume CBCT scans were taken with a Planmeca ProMax 3Ds scanner (Planmeca OY, Helsinki, Finland) with exposure parameters of 90 kV, 10 mA, and 12 s.

### 2.6. Evaluation Factors

Information on a number of factors (Table 1) was collected to assess the impact on treatment outcome.

### 2.7. Outcome Assessment

**Figure 1:**
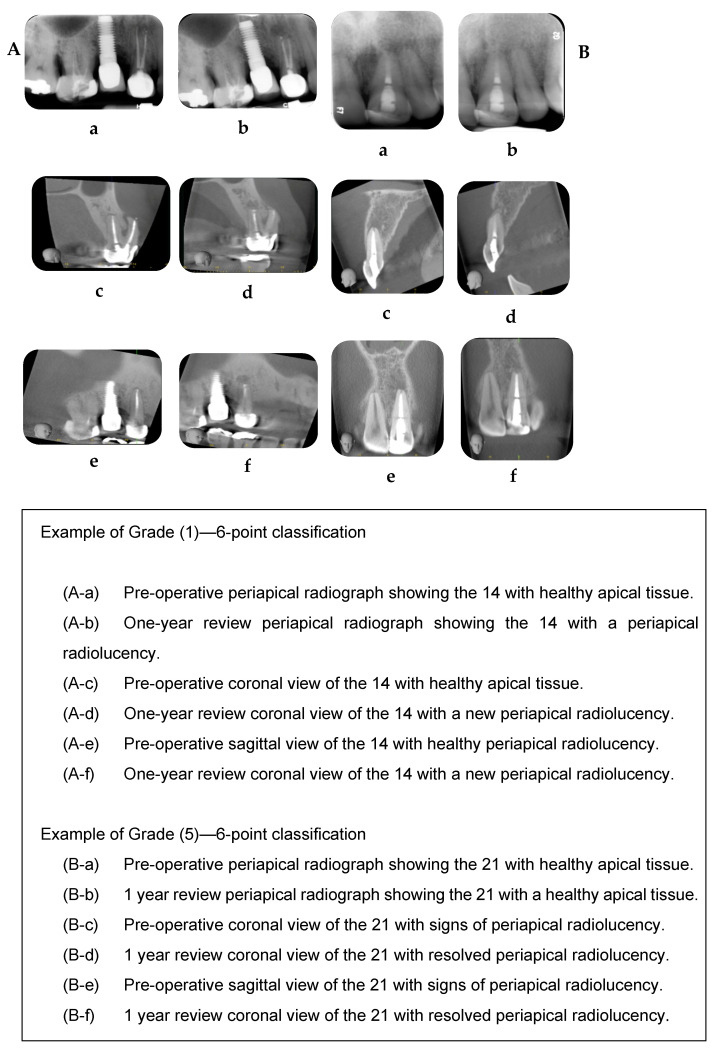
Implementation of 6-point classification criteria on study sample.

PR and CBCT scans were assessed by two experienced, calibrated endodontists who were not involved in the endodontic microsurgery procedure. CBCT images were chosen as a starting point for each root to be observed based on the images that best confirmed the presence or absence of an apical radiolucency in the sagittal, coronal, and/or axial planes. The CBCT volume was assessed using an i-Dixel-3DX version 1.8 software for the cases performed at Guy’s Hospital, and Planmeca Romexis software (https://www.planmeca.com/software/key-benefits/, accessed on 29 January 2024) was used for the cases performed in private practice. The CBCT volumes were also made available to the assessors in case more information was needed to determine healing. The PAs and CBCT slices were taken during pre-treatment and at 1 and 2 years post-treatment. The outcome assessment was undertaken using the classification proposed by Patel et al. [16] (Table 2). The outcome of the teeth was classified using both strict (healed) and loose (healing) criteria. The loose criteria (complete healing of apical tissues and reduction in size of apical radiolucencies) included grades 4, 5, and 6 as a favourable outcome, while the strict criteria (complete healing of apical tissues) included only grades 5 and 6. Figure 1 describes how the 6-point classification was implemented in the study sample. A consensus decision was reached for each of the radiographs and series of reconstructed CBCT images. An Excel (Excel 2022; Microsoft Corporation, Richmond, WA, USA) spreadsheet was created to log data. Statistical analyses were undertaken using IBM SPSS (Version 15.0). Kappa (k) index was used to assess the concordance between PA and CBCT. Furthermore, McNemar’s test was used to assess the asymmetry in concordance between PA and CBCT. Simple binary logistic regression models using GEE (generalised estimation equations) were performed to study the probability of unfavourable outcome according to independent variables. Non-adjusted odds ratio (OR) and 95% confidence intervals were obtained. Relevant variables (*p* < 0.1) were selected to enter a multiple model using the stepwise method. Adjusted ORs were obtained. The ROC curve, the corresponding AUC (area under the curve), and indexes and rates of diagnostic and predictive validity (sensitivity, specificity, PPV, NPV) were obtained in order to check the reliability of the model as a predictive tool. In order to detect differences in success rates between independent groups, a power of 92.2% was estimated for rates of 75–95% for 150 independent teeth at 95% confidence; the power was then corrected because of the within-subject dependence of observations. Considering a ratio of 1.2 teeth per patient and assuming a moderate intra-class correlation (ICC = 0.5), an effective power of 89.2% was estimated. Sixty-one teeth were re-assessed to establish inter-examiner reliability for both PA and CBCT; the linear Kappa (κ) index was used to assess the agreement between assessors (Table 3).

## 3. Results

A total of 150 teeth in 123 patients were assessed. The percentage of males and females was 43.1% and 56.9%, respectively, with over 50% of the patients aged 50–69 years. Of the cases, 68.7% (n = 102) were anterior, 16.7% (n = 25) were premolar, and 14.7% (n = 22) were molar teeth. Fifty-nine patients (39.3%) were reviewed at 1 year, 47 patients were reviewed at 2 years (31.3%), and 44 (29.3%) were reviewed at both 1 and 2 years.

**Figure 2:**
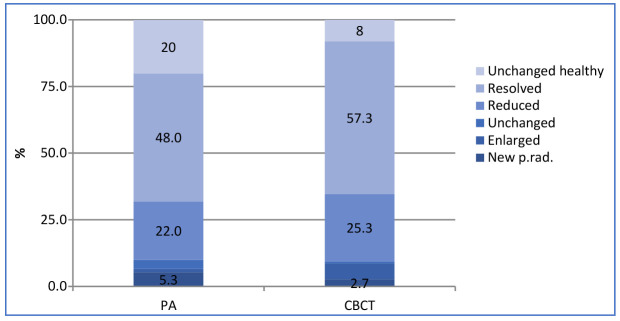
PA and CBCT diagnoses.

**Figure 3:**
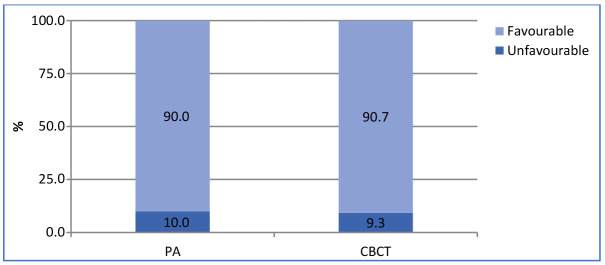
PA and CBCT success rates.

With PR, the healing and healed rate was 90% and 70%, respectively. When CBCT was used, the healing and healed rate was 90.6% and 82.6%, respectively (Figure 2 and Figure 3). Ninety-three teeth showed the same outcome with PR and CBCT. The agreement between the two techniques was moderate (k = 0.51).

**Figure 4:**
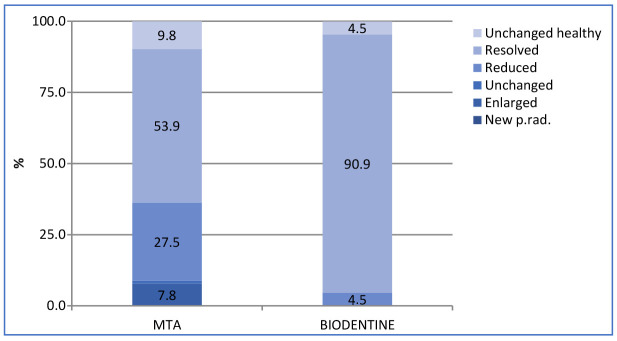
CBCT outcome by root-end filling material.

Using CBCT with strict criteria in the simple binary regression analysis, specialist operators were significantly associated with lower rates of unfavourable outcomes compared to postgraduate students (*p* = 0.005). Teeth that had received their initial root canal treatment by specialists had a significantly higher success rate than those initially treated by postgraduate students (*p* = 0.017). Biodentine reduced the odds of failure compared to MTA (OR = 0.08; *p* = 0.018) and IRM (*p* = 0.019). The failure rate with MTA, IRM, and Biodentine was 36.4%, 52.2%, and 4.5%, respectively (Figure 4). When a multiple regression model was constructed, including all the significant factors, Biodentine as a root-filling material remained a good prognostic factor (Table 4).

Using loose criteria with CBCT according to the multiple regression model, IRM as root-filling material (*p* = 0.013) and tooth mobility (*p* = 0.019) were found to be bad prognostic factors (Table 5). The results of the simple binary regression analysis for both PR and CBCT are reported in Table 6 and Table 7. 

The Association between PR status and independent variables using multiple binary logistic regression model using GEE for probability of unfavourable diagnosis are presented in Table 8.

## 4. Discussion

In this retrospective CBCT cohort study on the outcomes of surgical endodontics in a hospital referral centre and private practice, the success rate at 1–2 years was found to be very high (90%). In the multiregression analysis, the use of Biodentine was associated with a higher success rate using both loose and strict criteria, whereas tooth mobility had a negative effect on the outcome with a rate of 66.7%. However, these results should be interpreted with caution due to the low number of teeth presenting with mobility (n = 3).

The high success rate of surgical endodontics employing modern techniques such as operative microscopes, ultrasonic apical preparation, and MTA/calcium silicate cement as retrofilling material has been previously reported using periapical radiographs [17,18] and CBCT [19]. To the authors’ knowledge, this is the first apical surgery CBCT study in which different retrofilling materials have been compared. Considering that there was no significant difference between the three root-filling materials in terms of “healing” radiolucencies, the higher proportion of complete healings observed with Biodentine is likely to indicate a “faster healing” potential. This may well be associated with the intense production of calcium silicate hydrate and calcium hydroxide that has been observed with this calcium silicate cement [20], which is known to have a better seal than other materials when used in a liquid-rich environment, as addressed by previous studies [21]. To the authors’ knowledge, this is also the first independent clinical investigation on Biodentine as a retrofilling material. Biodentine is routinely used by many general dental practitioners and endodontists for its effectiveness in pulp capping and pulpotomy, open apices obturation [22,23,24], and perforation repair [25]. However, Biodentine is less popular as a retrofilling material due to its lower radiopacity compared to MTA and other calcium silicate materials [26].

Randomised trials with long-term follow-ups and larger sample sizes are warranted before reaching definitive conclusions.

In simple regression analyses, specialist operators were significantly associated with lower rates of unfavourable outcomes compared to postgraduate students, and teeth that had received their initial root canal treatment by specialists had a significantly higher success rate than those initially treated by postgraduate students. However, none of these factors remained significant in the multiregression analysis; this is not surprising considering the relatively small number of teeth and patients involved and the large number of uncontrolled variables inevitably associated with a retrospective clinical study.

The percentage of favourable outcomes was almost identical for CBCT and PA. However, only 93 out of 150 teeth showed the same outcome with PA and CBCT, with higher numbers of “resolved radiolucencies” detected with CBCT and a higher number of “unchanged healthy” teeth detected with PA. This is clearly due to the higher sensitivity of CBCT in detecting apical radiolucencies, as demonstrated in a histology study [27]. PA radiographs are the method of choice for the assessment of the outcome of surgical endodontics. The results obtained using PA radiographs were reported, as this may help in understanding to what extent periapical radiographs underestimate the presence of apical periodontitis in teeth endodontically treated and also in teeth treated with surgical endodontics.

The intra-examiner agreement was high with both imaging modality techniques, and both examiners were experienced in the use of PA and CBCT in the assessment of apical radiolucencies.

Most studies on pre-operative signs and symptoms have concluded that pre-operative pain and swelling were found to be the most significant pre-operative factors influencing the outcome of endodontic microsurgery [28,29,30,31]. It is possible that the large number of factors taken into consideration in our multiregression analysis, together with the relatively small number of teeth presenting with pain and swelling, may have reduced the impact of these factors on the outcome.

The main limitations of this study include its retrospective nature, the relatively small number of patients involved, and the large standard deviation associated with some of the statistically significant results in the multiregression analysis. It was our intention to include a much larger number of patients; however, this was not possible due to the reluctance of patients to visit the hospital and practice during the pandemic.

## 5. Conclusions

Endodontic microsurgery had a high healing rate regardless of the retrofilling material used. This is the first CBCT study comparing different retrofilling materials; within the limitations of this study, among the tested materials, Biodentine showed the highest percentage of completely healed radiolucencies.

## Figures and Tables

**Table 1:** Evaluation factors.

1. Patient age
2. Patient gender
3. Patient medical history
4. Patient social history
5. Tooth type
6. Tooth position
7. Type of initial treatment
8. Age of initial treatment
9. Operator of initial treatment
10. Diagnosis before root-end surgery
11. Presence or absence of an apical crack
12. Presence or absence of parafunctional habits
13. Presence of pre-operative signs and symptoms
14. Presence of radiographic pre-operative radiolucency
15. Root-end surgery operator
16. Amount of root-end preparation
17. Type of root-end material
18. Histology of apical tissues
19. Presence or absence of a post
20. Type of post
21. Root canal length
22. Root canal quality

**Table 2:** Six-point classification.

Grade	Interpretation
1	New periapical radiolucency
2	Enlarged periapical radiolucency
3	Unchanged periapical radiolucency
4	Reduced periapical radiolucency
5	Resolved periapical radiolucency
6	Unchanged healthy periodical status (no radiolucency before and after (re)treatment)

**Table 3:** Intra-examiner concordance in 6-category outcomes using CBCT and PA: weighted Kappa’s concordance index and 95% confidence interval.

Imaging Method	Kappa	95% CI	Assessment
PA	0.78	0.66–0.90	Substantial/Very good
CBCT	0.81	0.67–0.95	Substantial/Very good

**Table 4:** Association between CBCT status and independent variables when strict criteria was used: results of multiple binary logistic regression model using GEE for the probability of an unfavourable diagnosis. Adjusted OR and 95% confidence interval.

	Category	OR	CI 95%	*p*-Value
Operator	PG	1		
	SP	0.42	0.03–5.94	0.519
Initial treatment operator	PG	1		0.622
	SP	2.78	0.18–42.5	0.462
	GDP	3.24	0.26–39.8	0.358
Root-end filling	MTA	1		0.084
	Biodentine	0.09	0.01–0.79	0.030 *
	IRM	1.08	0.35–3.34	0.896
	Bioceramic putty	--	--	--
Tender to palpation	No	1		
	Yes	1.15	0.39–3.44	0.801

* *p* < 0.05.

**Table 5:** Association between CBCT status and independent variables using loose criteria: results of multiple binary logistic regression model using GEE for the probability of unfavourable diagnosis. Adjusted OR and 95% confidence interval.

	Category	OR	CI 95%	*p*-Value
Initial treatment	RCT	1		0.471
	Re-RCT	0.82	0.23–2.87	0.751
	Surgery	4.07	0.36–46.2	0.258
	No RCT	--	--	--
Root-end filling	MTA	1		
	Biodentine	--	--	--
	IRM	4.33	1.37–13.7	0.013 *
	Bioceramic putty	--	--	--
Mobility	No	1		
	Yes	21.2	1.65–271.1	0.019 *

* *p* < 0.05.

**Table 6:** Association between PA status and independent variables: results of simple binary logistic regression model using GEE for the probability of unfavourable diagnosis. Non-adjusted OR and 95% confidence interval.

	Category	OR	CI 95%	*p*-Value
Gender	Male	1		
	Female	3.11	0.84–11.5	0.645
Age group	<40 years	1		0.573
	40–49 years	2.76	0.23–33.2	0.423
	50–59 years	6.04	0.66–55.7	0.112
	60–69 years	3.54	0.40–31.5	0.258
	>70 years	2.90	0.24–35.5	0.406
Operator	PG	1		
	SP	0.78	0.25–2.47	0.677
Review dates	12 months	1		0.967
	24 months	1.05	0.31–3.61	0.936
	12 and 24 months	0.88	0.23–3.35	0.855
Tooth type	Anterior	1		0.052
	Premolar	4.04	1.14–14.3	0.030 *
	Molar	3.59	0.93–13.9	0.064
Arch	Maxilla	1		
	Mandible	1.58	0.31–7.94	0.581
Histology	None	1		0.476
	Granuloma	0.44	0.05–3.64	0.446
	Cyst	0.36	0.05–2.66	0.314
Root	M/MB	1		0.933
	M + D	1.13	0.12–10.2	0.917
	B/P/B + P	1.50	0.17–13.3	0.716
Initial treatment	RCT	1		0.086
	Re-RCT	1.90	0.58–6.23	0.293
	Surgery	8.08	1.18–55.2	0.033 *
	No RCT	--	--	--
Initial treatment operator	PG	1		0.724
	SP	0.53	0.09–3.08	0.483
	GDP	0.99	0.28–3.49	0.983
RCT age	0–5 years	1		0.481
	6–10 years	--	--	--
	11–20 years	2.21	0.58–8.43	0.246
	>20 years	1.93	0.33–11.2	0.465
Diagnosis	CAP with previous filling	1		
	Others	0.50	0.06–4.04	0.512
Apical crack	No	1		
	Yes	6.07	0.49–75.5	0.161
Root-end preparation	3 mm	1		
	>3 mm	0.64	0.17–2.39	0.506
Root-end filling	MTA	1		0.029 *
	Biodentine	1.36	0.26–7.16	0.719
	IRM	4.79	1.49–15.4	0.009 **
	Bioceramic putty	--	--	--
Pain	No	1		
	Yes	--	--	--
Tender to percussion	No	1		
	Yes	1.87	0.53–6.53	0.328
Tender to palpation	No	1		
	Yes	1.10	0.29–4.25	0.890
Sinus	No	1		
	Yes	1.55	0.49–4.86	0.456
Swelling	No	1		
	Yes	0.31	0.04–2.51	0.275
Discharge	No	1		
	Yes	--	--	--
Mobility	No	1		
	Yes	20.6	1.75–242.4	0.016 *
Probing	No	1		
	Yes	--	--	--
Recession	No	1		
	Yes	1.86	0.20–17.0	0.584
Medical history	Fit and healthy	1		
	Compromised	4.53	1.38–14.9	0.013 *
Smoking	No	1		
	Yes	--	--	--
Post	No	1		
	Yes	1.23	0.43–3.53	0.699
Post type	M	1		
	F	0.44	0.05–3.82	0.455
Pre-operative signs and symptoms	No	1		
	Yes	0.79	0.27–2.26	0.654
Lesion	No	1		
	Yes	--	--	--
Root canal length	Adequate	1		
	Short	0.51	0.16–1.69	0.513
	Long	--	--	--
Root canal quality	Adequate	1		
	Inadequate	1.60	0.53–4.84	0.410

* *p* < 0.05; ** *p* < 0.01.

**Table 7:** Association between CBCT status and independent variables: Results of simple binary logistic regression model using GEE for the probability of an unfavourable diagnosis. Non-adjusted OR and 95% confidence interval.

	Category	OR	CI 95%	*p*-Value
Gender	Male	1		
	Female	0.94	0.45–1.95	0.869
Age group	<40 years	1		0.557
	40–49 years	1.54	0.47–5.04	0.477
	50–59 years	1.63	0.53–4.94	0.392
	60–69 years	0.79	0.26–2.39	0.674
	>70 years	0.75	0.23–2.47	0.637
Operator	PG	1		
	SP	0.32	0.15–0.71	0.005 **
Review dates	12 months	1		0.105
	24 months	1.72	0.73–4.04	0.216
	12 and 24 months	0.57	0.24–1.37	0.209
Tooth type	Anterior	1		0.233
	Premolar	1.14	0.45–2.87	0.780
	Molar	0.38	0.12–1.24	0.109
Arch	Maxilla	1		
	Mandible	1.47	0.51–4.21	0.476
Histology	None	1		0.693
	Granuloma	1.64	0.51–5.29	0.404
	Cyst	1.18	0.43–3.19	0.752
Root	M/MB	1		0.085
	M + D	4.29	0.36–51.8	0.252
	B/P/B + P	16.7	1.32–210.1	0.030 *
Initial treatment	RCT	1		0.385
	Re-RCT	1.74	0.77–3.92	0.183
	Surgery	3.42	0.54–21.7	0.192
	No RCT	1.14	0.07–18.9	0.927
Initial treatment operator	PG	1		0.051
	SP	0.28	0.10–0.80	0.017 *
	GDP	0.49	0.21–1.15	0.102
RCT age	0–5 years	1		0.678
	6–10 years	0.61	0.18–2.09	0.429
	11–20 years	0.58	0.22–1.56	0.580
	>20 years	0.95	0.27–3.31	0.937
Diagnosis	CAP with previous filling	1		
	Others	0.94	0.29–3.04	0.911
Apical crack	No	1		
	Yes	1.42	0.12–16.4	0.780
Root-end preparation	3 mm	1		
	>3 mm	0.71	0.31–1.67	0.436
Root-end filling	MTA	1		0.019 *
	Biodentine	0.08	0.01–0.66	0.018 *
	IRM	1.92	0.73–5.02	0.185
	Bioceramic putty	--	--	--
Pain	No	1		
	Yes	1.92	0.26–14.1	0.522
Tender to percussion	No	1		
	Yes	0.81	0.33–1.98	0.640
Tender to palpation	No	1		
	Yes	2.21	0.98–4.97	0.055
Sinus	No	1		
	Yes	1.14	0.49–2.63	0.766
Swelling	No	1		
	Yes	1.49	0.61–3.61	0.384
Discharge	No	1		
	Yes	0.46	0.08–2.62	0.382
Mobility	No	1		
	Yes	3.88	0.34–44.0	0.274
Probing	No	1		
	Yes	--	--	--
Recession	No	1		
	Yes	0.37	0.04–3.22	0.364
Medical history	Fit and healthy	1		
	Compromised	1.35	0.65–2.84	0.423
Smoking	No	1		
	Yes	1.27	0.31–5.22	0.743
Post	No	1		
	Yes	0.86	0.43–1.71	0.663
Post type	M	1		
	F	0.55	0.16–1.87	0.342
Pre-operative signs and symptoms	No	1		
	Yes	1.20	0.57–2.54	0.634
Lesion	No	1		
	Yes	--	--	--
Root canal length	Adequate	1		0.181
	Short	1.41	0.69–2.91	0.350
	Long	0.22	0.03–1.88	0.166
Root canal quality	Adequate	1		
	Inadequate	0.94	0.46–1.91	0.864

* *p* < 0.05; ** *p* < 0.01.

**Table 8:** Association between PA status and independent variables: results of multiple binary logistic regression model using GEE for probability of unfavourable diagnosis. Adjusted odds ratio (OR) and 95% confidence interval.

	Category	OR	CI 95%	*p*-Value
Tooth type	Anterior	1		0.068
	Premolar	8.47	1.17–61.4	0.035 *
	Molar	4.13	0.83–20.7	0.084
Initial treatment	RCT	1		0.085
	Re-RCT	2.50	0.63–9.89	0.193
	Surgery	19.4	1.42–262.4	0.026 *
	No RCT	--	--	--
Root-end filling	MTA	1		0.044 *
	Biodentine	3.37	0.32–36.1	0.315
	IRM	7.69	1.48–40.1	0.015 *
	Bioceramic putty	--	--	--
Mobility	No	1		
	Yes	34.1	2.41–482.3	0.009 **
Medical history	Fit and healthy	1		
	Compromised	10.4	1.11–98.0	0.040 *

* *p* < 0.05; ** *p* < 0.01.

## Data Availability

Data are contained within the article and Supplementary Materials.

## Supplementary Materials

The following supporting information can be downloaded at: https://www.mdpi.com/article/10.3390/jcm13061692/s1.
